# Further Observations on the Results of Blood Transfusion in War Surgery

**Published:** 1918-04

**Authors:** 


***Further Observations on the Results of Blood Transfusion in War Surgery.*** L. Bruce Robertson, M. B., Major C. A. M. C, and Col. C. Gordon Watson. Abs. from the *Annals of Surgery January*, 1918.

R. describes 36 cases of transfusion with results which he classifies as follows : Life saving, 22 ; immediately beneficial but died from infection or shock, 9; no benefit, 3; harmful, 2. Though the mortality was comparatively high the author states that all the patients were in a desperate condition, and, with perhaps one exception, could not have been expected to survive if transfusion had been withheld. His experience showed that when the post-operative condition was one of progressively increasing shock, blood transfusion was a permanent resuscitative measure of extreme value.

Clinical observation appears to show that some degenerative changes take place in the organism when the exsanguinated condition persists for more than a few hours. For this reason it is advisable to give the blood as soon after admission as circumstances permit.

In cases of severe primary hemorrhage 700 to 1000 cc. of blood will usually tide the patient over his crisis. The transfusion should not be too rapid, and the intervals between the injections of the blood should be longer towards the latter part than at the beginning of the procedure.

Blood-pressure readings before and after blood-transfusion showed that the rise was well maintained; this fact is in marked contrast to the transient effect on the blood-pressure of normal saline injections.

In two cf the author's cases hemolysis occurred — in one of these the citrate method was used.

All the cases are given in detail.

W. confirms these observations and states that the results from blood transfusion have been infinitely better than those formerly obtained by saline infusion.

He reports comparatively few unfavorable results, but warns against the danger of giving blood too rapidly or in too large quantities, especially if the heart is already tired.

